# Herpes zoster in lupus nephritis: experience on 292 patients followed up for 15 years

**DOI:** 10.3389/fimmu.2023.1293269

**Published:** 2023-11-22

**Authors:** Francesco Reggiani, Silvia Cardi, Fabio Tumminello, Marta Calatroni, Laura Locatelli, Maria Gerosa, Nicoletta Del Papa, Gabriella Moroni

**Affiliations:** ^1^ Department of Biomedical Sciences, Humanitas University, Milan, Italy; ^2^ Nephrology and Dialysis Unit, IRCCS Humanitas Research Hospital, Milan, Italy; ^3^ Department of Clinical Sciences and Community Health, University of Milan, Milan, Italy; ^4^ Clinical Rheumatology Unit, ASST Pini-CTO, Milan, Italy

**Keywords:** herpes zoster, systemic lupus erythematosus, lupus nephritis, immunosuppressive therapy, vaccination

## Abstract

**Objectives:**

To evaluate the prevalence, incidence, and predictors of herpes zoster (HZ) development in lupus nephritis (LN).

**Methods:**

This retrospective study included 292 LN patients to determine HZ incidence during the last decades and its correlation with LN activity. LN patients with HZ were matched with LN patients without HZ in a 1:2 ratio based on sex, age, year of LN diagnosis, and LN histological class at kidney biopsy to assess HZ risk factors. Statistical tests included t-test, U-test, and Fisher’s test. Univariate and multivariate logistic regression analyses were conducted to identify potential risk factors.

**Results:**

HZ occurred after LN diagnosis in 66 patients (prevalence 22.6%) with an average of 8.7 years (range 0.2–28.4 years). Although with the potential limitations of the retrospective nature and the extensive duration of the study, the incidence of HZ was 15.6/1,000 person-years, increasing from 6.9 before 1980 to 16.0 in the 1990s and 43.9 after 2010. HZ onset was unrelated to LN activity. LN was active in 43% of cases and quiescent in the other 57% of cases at HZ diagnosis. The percentage of patients who developed lupus flares during the year after HZ (18.9%) was not different from that which occurred during the year before HZ (17.2%, p = 0.804). After excluding confounding factors through matching, the univariate analysis suggested that cyclosporin during induction therapy (p = 0.011) and higher cumulative doses of glucocorticoids (GCs; >50 g, p = 0.004), cyclophosphamide (CYC; >5 g, p = 0.001), and mycophenolate mofetil (MMF > 1,000 g, p = 0.007) predisposed patients to HZ. Univariate and multivariate analyses revealed a protective role of azathioprine (p = 0.008) and methylprednisolone pulses (p = 0.010) during induction therapy.

**Conclusions:**

HZ occurs unpredictably throughout the course of LN, underscoring the importance of continuous monitoring for these patients. In addition, the incidence of HZ seems to have increased in recent decades. Induction therapy with azathioprine and methylprednisolone pulses appears to provide protection, while higher cumulative doses of GCs, CYC, and MMF increase susceptibility.

## 1 Introduction

Lupus nephritis (LN) is the most frequent and severe organ manifestation of systemic lupus erythematosus (SLE) (1). The spectrum of clinical presentation of LN is wide, ranging from silent urinary abnormalities to rapidly progressive renal failure, and the clinical course is characterized by phases of remission alternated by renal or extrarenal reactivation of SLE, called flares (2). The treatment almost always consists of glucocorticoids (GCs) and immunosuppressants (ISs), such as mycophenolate mofetil (MMF) and cyclophosphamide (CYC), and recently biological drugs were also employed (3).

Infections, triggered by immunosuppressive treatments and the impaired immune system during active LN, were in the past a substantial cause of mortality but today represent a concern (4, 5). Frequent bacterial infections include urinary tract infections, pneumonia, and bacteremia without known focus (6).

Herpes zoster (HZ) is considered one of the most frequent viral infections in patients with SLE (7, 8). Varicella zoster virus (VZV), or human herpes virus 3 (HHV3), is a neurotropic human herpes virus belonging to the genus *Alphaherpesvirinae*. VZV is distributed worldwide and is responsible for varicella (chickenpox) and herpes zoster (shingles) (9). Varicella is the clinical presentation of primary infection and usually infects young individuals. HZ represents a reactivation of VZV in the host and is typically characterized by the appearance of painful inflammation and vesiculation in the dermatome correlated to the ganglionic neurons in which the VZV has become latent after primary infection (10). However, the HZ clinical picture is variable, and some complications may occur, such as myelitis, cranial nerve palsies, meningitis, vasculopathy, retinitis, and gastroenterological infections (11). The incidence of HZ in the general population ranges from 1.2 to 4.9 cases per 1,000 person-years (12, 13). VZV reactivation is triggered by a reduction of cell-mediated immunity, as it occurs in advancing age or immunosuppressive states (cancer, human immunodeficiency virus, and bone marrow or solid organ transplantation and during immunosuppressive therapy in autoimmune diseases) (11).

The epidemiological data on HZ in SLE are scanty and contradictory. In the literature, the prevalence ranges from 3.6% to 30.5%, and the incidence is between 6.4 and 37.7 per 1,000 person-years (7, 14–18). These discrepancies may be attributed to the different sample sizes and ethnicity of patients and the duration of the observation period. Moreover, HZ diagnosis is still nowadays a clinical diagnosis, with the risk of underdiagnosis or misdiagnosis.

Data on HZ in LN are even more scanty. In a retrospective study on 251 Chinese patients with LN, 55 episodes of HZ were observed during the first 2 years after LN diagnosis, determining a prevalence of 18.0% and an incidence of 8.84 per 100 person-years (8).

The timing of HZ onset in relation to SLE activity is not defined. Chen et al. demonstrated a higher risk of HZ within 3–6 months following SLE diagnosis (18), while in the study by Kwan et al., a significant number of cases occurred more than 10 years after SLE diagnosis (7).

To the best of our knowledge, there are no studies in LN that have evaluated the change in the incidence of HZ in LN over the last decades, as well as the relationship between the phase of clinical activity of LN and HZ onset.

To cover this lack of knowledge, we have performed this study with the aims: a) to investigate the changes in the epidemiology of HZ in LN during the last decades, b) to evaluate the correlation between HZ occurrence after LN diagnosis and the phases of clinical activity of LN, c) to establish the impact of HZ on subsequent renal flares and chronic kidney disease (CKD) development, and d) to identify potential risk factors for HZ in LN population.

## 2 Materials and methods

We retrospectively analyzed the outcome of 292 LN patients followed up at IRCCS Humanitas Research Hospital and included them in the LN database from 1970 onwards. Inclusion criteria were age > 18 years, diagnosis of SLE according to American College of Rheumatology (ACR) criteria (19), and the presence of clinical or biopsy-proven LN. Exclusion criteria were a follow-up shorter than 12 months and the presence of incomplete clinical records.

The diagnosis of HZ was based on the history and physical findings and confirmed by an expert physician. First, we compared the clinical and histological presentation, as well as the therapy administered at the time of LN diagnosis, between patients who subsequently developed HZ and those who did not experience HZ. Then, patients with HZ (cases) were matched with a subgroup (controls) chosen according to sex, age (±5 years) at diagnosis of LN, year of LN diagnosis (±5 years), and histological class at kidney biopsy in a 1:2 case–control ratio.

The cumulative dose of GCs and ISs was calculated by adding the partial doses of the intravenous administration to the partial doses produced by each of the different periods during which the patient received drugs orally. For GCs, the cumulative dose is expressed as the total prednisone-equivalent dose in grams.

The study was approved by the Ethics Committee of IRCCS Humanitas Research Hospital, Milan, Italy (protocol code NEF0032023). All patients signed an informed consent for the scientific use of their data that were anonymized.

### 2.1 Definitions

SLE: classified according to the 2019 European League Against Rheumatism/American College of Rheumatology Classification Criteria for Systemic Lupus Erythematosus (19). All patients diagnosed before 2019 were reclassified based on ACR 2019 criteria (19).

LN: diagnosed on clinical grounds or with kidney biopsies. Kidney biopsies were classified according to the 2003 International Society of Nephrology/Renal Pathology Society (ISN/RPS) criteria, revised in 2018 (20, 21). Renal biopsies performed before 2002 were reclassified according to the last ISN/RPS classification (22). Class II and V were considered “non-proliferative forms”, while III and IV associated or not with class V were considered “proliferative forms”. Chronicity index (CI) and activity index (AI) were assessed according to Austin et al. (21, 23). In patients in which renal biopsy was not feasible, LN diagnosis was defined as impaired kidney function and/or urinary signs of renal involvement not explained by causes other than LN in the presence of a confirmed SLE diagnosis.

Normal kidney function: serum creatinine ≤1 mg/dL and estimated glomerular filtration rate (eGFR) >60 mL/min/1.73 m^2^ by the Chronic Kidney Disease Epidemiology Collaboration (CKD-EPI) equation.

Complete renal remission: normal kidney function, inactive urinary sediment (<5 red blood cells per higher power field and no erythrocyte casts) and proteinuria ≤0.5 g/day. *Partial renal remission:* ≥50% reduction in proteinuria to sub-nephrotic levels and normal or near normal eGFR (24).

CKD: GFR < 60 mL/min per 1.73 m^2^ by the CKD-EPI equation or markers of kidney damage for >3 months (25).

Proteinuria: measured by benzethonium chloride on the urine collected over 24 hours expressed as g/day.

SLE flare: defined as an increase in disease activity, both extrarenal and renal, requiring changes or intensification of therapy (26). Renal flares were defined as follows:

- *Nephritic flare:* increase in serum creatinine of at least 30% over the last value associated with nephritic urinary sediment, with or without increased proteinuria (27).
- *Proteinuric flare:* increase in proteinuria of at least 2 g/day if the previous proteinuria was <3.5 g/day or a doubling if the previous proteinuria was ≥3.5 g/day (27).

HZ therapy: according to best practice guidelines, HZ therapy consisted of acyclovir or valaciclovir (28). Dose and duration were determined considering the severity of the infection and renal function.

### 2.2 Statistical analysis

Demographic and clinical data are expressed as numbers or percentages in the case of discrete variables, whereas in the case of continuous variables, they are expressed as median and interquartile range (IQR) or average ± standard deviation (SD), where appropriate. The demographic characteristics were analyzed using Student’s t-test, the Mann–Whitney U-test, and Fisher’s test, where appropriate. Univariate and multivariate logistic regression models, testing clinically and histologically relevant variables, were performed to identify potential risk factors for HZ reactivation. The Kaplan–Meier estimate was used to draw survival curves, and the Mantel–Cox log-rank test was used to test their difference. Statistical significance was defined as p < 0.05. Data were analyzed using STATA version 16.1 (StataCorp).

## 3 Results

### 3.1 Patient cohort

A total of 292 patients were enrolled in this study (**Table 1**), of which 89% were female and 267 (91.4%) were White. The median duration of SLE before the diagnosis of LN was 3.4 years (IQR 0–4.4). Clinical presentation at the onset of LN encompassed normal renal function in 121 (41.4%) patients, nephrotic syndrome in 145 (49.7%), and arterial hypertension in 145 (49.7%).

**Table 1:** Demographic and clinical characteristics of the lupus nephritis cohort.

	All patients (n = 292)
**Duration of SLE before LN diagnosis (months)**	3.4 (IQR 0–4.4)
**Age at lupus nephritis diagnosis (years)**	28.9 (IQR 21.3–39.1)
**Sex (female)**	89.0% (260/292)
Histological Class	
Proliferative forms (III and IV)	76.0% (209/275)
Non-proliferative forms (II and V)	23.6% (65/275)
Class VI	0.4% (1/275)
**Activity index**	6 (IQR 3–9)
**Chronicity index**	1 (IQR 0–3)
**Creatinine (mg/dL)**	0.9 (IQR 0.7–1.3)
**eGFR (mL/min)**	78.7 (IQR 51.3–109.0)
**eGFR > 60 mL/min and creatinine ≤ 1 mg/dL**	41.4% (121/292)
**Arterial hypertension**	50.2% (145/289)
**Proteinuria (g/day)**	3.4 (IQR 2.0–5.4)
**Nephrotic syndrome (proteinuria > 3.5 g/day)**	145 (IQR 49.66)
**Serum albumin (g/dL)**	2.9 (IQR 2.4–3.4)
**WBC (n/mmc)**	5700 (IQR 4000–7500)
**Hemoglobin (g/dL)**	11.2 (IQR 9.5–12.3)
**Platelets (10^3^/μL)**	228 (IQR 174–299)
**C3 (mg/dL)**	58 (IQR 47–78)
**C4 (mg/dL)**	10 (IQR 5–14)
**Anti-DNA positivity**	99.7% (291/292)
**Antiphospholipid antibody positivity**	36.8% (93/253)
**ENA (anti-SSA, anti-SSB, anti-SM, and/or anti-RNP) positivity**	56.5% (130/230)
Induction glucocorticoids	
Methylprednisolone pulses	82.8% (226/273)
Oral glucocorticoids	17.2% (47/273)
**Hydroxychloroquine at induction**	23.6% (69/292)
Induction immunosuppressants	
Cyclophosphamide	49.3% (112/227)
Azathioprine	13.2% (30/227)
Mycophenolate	23.4% (53/227)
Cyclosporin	4.8% (11/227)
Rituximab	9.3% (21/227)
**GC cumulative dose (g)**	26.5 (IQR 11.9–53.9)
**GC grams for year of follow-up**	2.1 (IQR 1.0–4.8)
**Follow-up (years)**	15 (IQR 6.1–24.9)
**CKD at last observation**	18.5% (54/289)
**Deaths at last observation**	13.7% (40/292)

If not differently specified, data are expressed as median and interquartile ranges.

C3, complement factor 3; C4, complement factor 4; CKD, chronic kidney disease; GCs, corticosteroids; ENA, extractable nuclear antigen (anti-SSA [anti-Sjögren’s-syndrome-related antigen A autoantibodies], anti-SSB [anti-Sjögren’s-syndrome-related antigen A autoantibodies], anti-SM [anti-Smith antibodies], and anti-RNP [anti-negative antinuclear ribonucleoprotein antibodies]); WBC, white blood cells; SLE, systemic lupus erythematosus; LN, lupus nephritis; eGFR, estimated glomerular filtration rate.

Seventeen patients (5.8%) were not subjected to kidney biopsy. In 275 patients (94.2%), renal biopsy revealed 62 cases of class III or III+V, 147 cases of class IV or class IV+V, 55 cases of pure membranous LN (class V), 10 cases of class I or II LN, and one case of class VI.

At baseline, 226 patients received intravenous methylprednisolone pulses (MP pulses) in three subsequent days, followed by prednisone 0.5 mg/kg/day for 4 weeks, and then tapered to 7.5–5 mg/day. Another 47 patients were treated with prednisone 1 mg/kg/day for 4 weeks, and then the dosage was progressively tapered. In addition to GCs, immunosuppressive drugs were used in 227 (77.7%) of patients as induction therapy. None of the patients were treated with JAK inhibitors anifrolumab or baricitinib. The only biologic drug used was rituximab (RTX). Hydroxychloroquine was taken by 69 patients (23.6%) at baseline and in another 38% of patients during the follow-up.

After a median follow-up of 15.0 (IQR 6.1–24.9) years, 54 patients (18.7%) developed CKD. Of them, 19 (6.5%) developed end-stage kidney disease (ESKD), and 40 (13.7%) patients died.

None of the patients received HZ vaccination. At least one episode of HZ reactivation was present after LN diagnosis in the medical history of 66 patients (22.6%). In **Supplementary Table 1**, a comparison between the characteristics of patients who developed or did not develop HZ is reported.

### 3.2 Severe forms of HZ

Only two out of the 66 patients (3%) developed a severe form of HZ that required hospitalization. A 60-year-old woman presented with cutaneous HZ in the gluteal region 3 years after LN diagnosis and subsequently developed transverse myelitis at the D6–D7 level with a motor deficit in the right lower limb. HZ myelitis was confirmed by polymerase chain reaction (PCR) for varicella zoster virus in the cerebrospinal fluid. The patient was receiving treatment with 15 mg/day of prednisone (cumulative dose 6.9 g) and 1.5 g/day of MMF (cumulative dose 689.0 g). Hydroxychloroquine was not prescribed due to the presence of glucose-6-phosphate dehydrogenase deficiency. For HZ, she was treated initially with valaciclovir and shifted to acyclovir after the diagnosis of transverse myelitis. Despite the resolution of HZ reactivation, the patient had residual difficulty in walking. The second patient was a 27-year-old woman; 11 months after the diagnosis of LN, she presented with widespread and extensive cutaneous HZ involvement in the right lower limb that required hospitalization due to functional impotence of the right leg accompanied by paresthesia. The patient was receiving treatment with 20 mg/day of prednisone (cumulative dose 3.3 g), 2.0 g/day of MMF (cumulative dose 631.5 g), and 200 mg/day of hydroxychloroquine. Of note, 2 months after HZ, an IgG-Kappa monoclonal gammopathy of low-intermediate risk developed. In all the other patients, the clinical course of HZ was not complicated. During the acute HZ phase, MMF was withdrawn in both patients.

### 3.3 Prevalence and incidence of HZ during the last five decades

**Figure 1:**
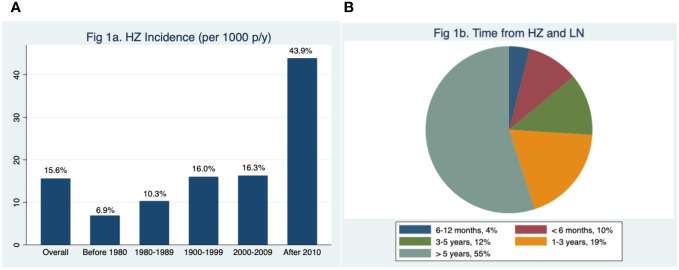
Incidence through decades of herpes zoster (HZ) reactivation in lupus nephritis (LN) **(A)**. Time from LN diagnosis and first HZ reactivation episode. p/y, person-year **(B)**.

The prevalence of HZ in our cohort was 22.6%, and the incidence was 15.6 per 1,000 person-years, increasing from 6.9 before 1980 to 16.0 in the 1990s and 43.9 after 2010 (**Figure 1A**). The average time between LN diagnosis and HZ was 8.7 years (IQR 1.8–14.7), with 14% of patients developing HZ within 1 year after the LN diagnosis (10% within 6 months), 19% between 1 and 3 years, 12% between 3 and 5 years, and the remaining 55% after 5 years from the LN diagnosis (**Figure 1B**).

### 3.4 Relationship between HZ activity and outcome of LN

**Figure 2:**
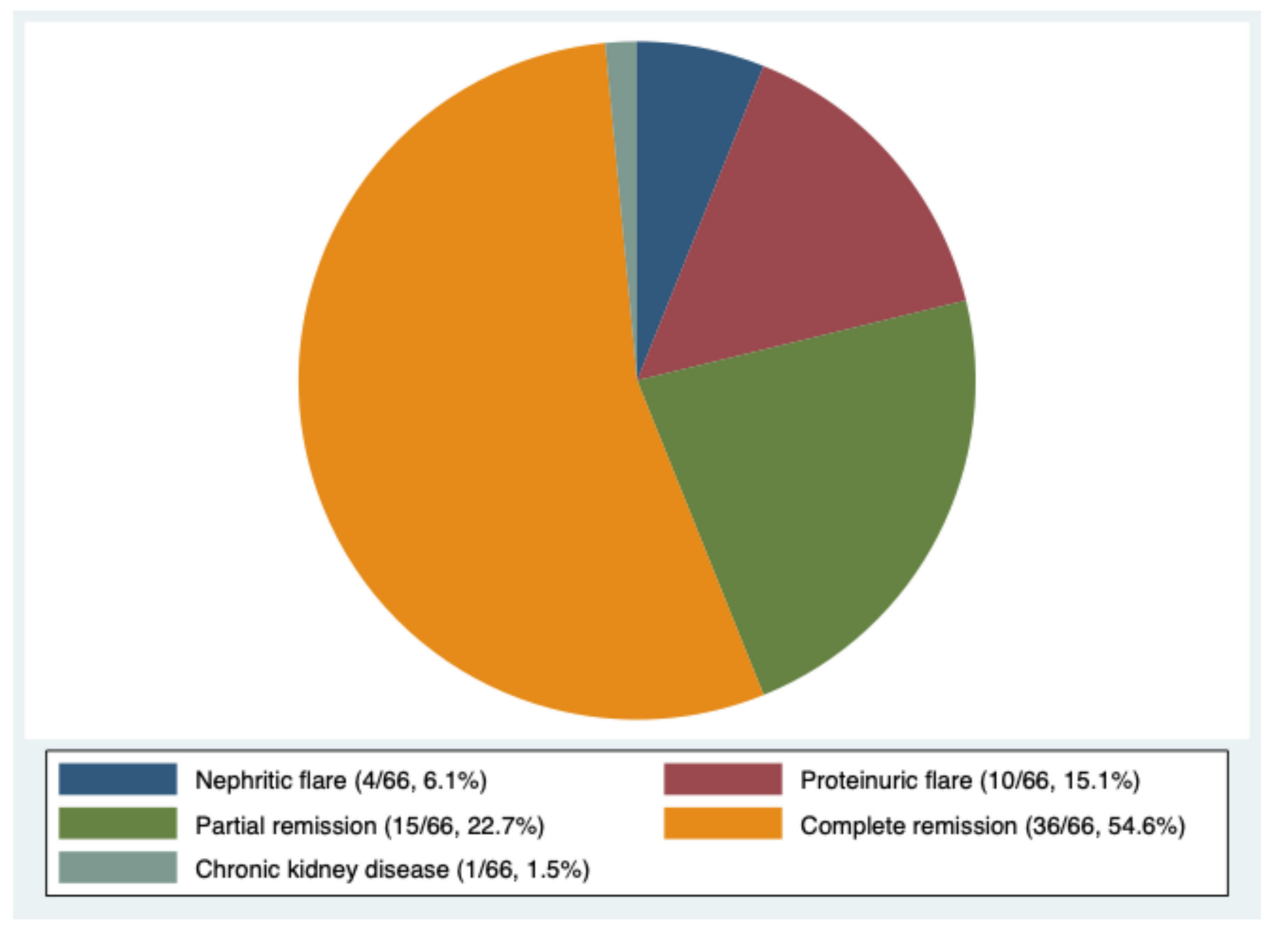
Correlation between lupus nephritis phases and herpes zoster reactivation (percentage of patients).

**Figure 2**, which reports the occurrence of HZ in the different phases of LN activity, demonstrates that the infection can develop both during active and quiescent LN. Out of the 66 patients with at least one episode of HZ, 29 patients (43.9%) developed HZ during an active phase of the disease (4 [6.1%] during a nephritic flare, 10 [15.1%] during a proteinuric flare, and 15 [22.7%] during partial remission of LN), 36 patients (54.6%) during complete remission, and one (1.5%) after the development of CKD. Considering the ongoing LN therapies at the time of HZ infection (**Supplementary Table 2**), the percentage of patients taking GCs did not differ between patients in the active phase and patients in remission (89.7% *vs.* 78.4%, p = 0.224), while the GC dose was significantly higher in patients in the active phase (prednisone 11.5 ± 7.0 *vs.* 6.2 ± 6.3 mg, p = 0.001). No significant difference was observed in the percentage of patients taking hydroxychloroquine or ISs.

The development of HZ does not seem to have worsened the renal outcome of LN. During the first year following HZ reactivation, 18.9% of patients in complete or partial remission experienced a renal flare. However, this rate of flare is not different from that observed during the year before HZ (17.2%, p = 0.804). At the end of the observation of a median of 15.0 years (IQR 6.1–24.9), 12.1% of patients who developed and 20.6% of those who did not develop HZ had CKD (p = 0.08). No difference was observed in mortality, which was overall 13.7% (13.6% in cases and 13.7% in controls, p = 1.00).

### 3.5 Comparison between patients who developed HZ (cases) and matched control group (controls)

**Figure 3:**
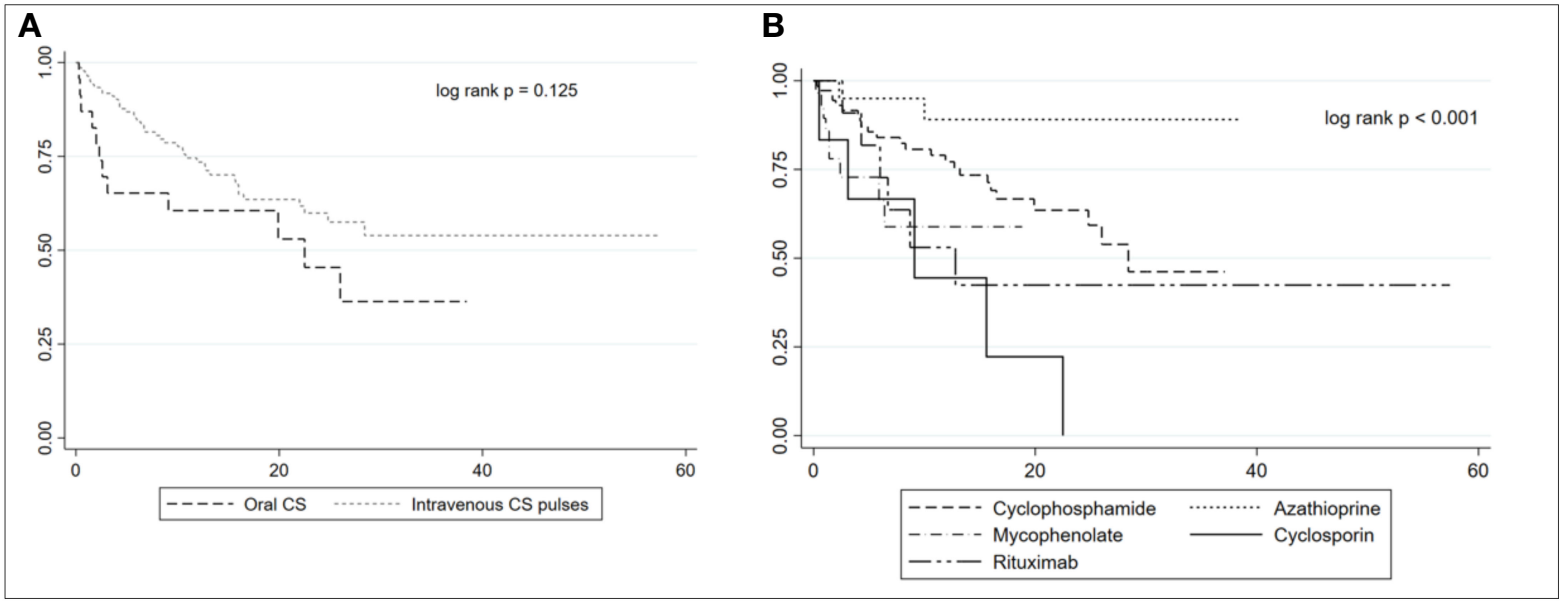
Kaplan–Meier plot of herpes zoster reactivation in patients treated with oral corticosteroids (CSs) or intravenous CS pulses **(A)**. Kaplan–Meier plot of herpes zoster reactivation according to the immunosuppressants used at induction **(B)**.

**Table 2** shows the demographic and clinical characteristics of patients after the matching. We did not observe significant differences in the initial clinical presentation and the activity and chronicity indexes at kidney biopsy between the two groups. We found a trend of more frequent use of oral GCs and less frequent use of MP pulses as induction therapy in HZ patients in comparison to controls (p = 0.106). Moreover, patients treated with oral GCs at induction tended to develop HZ reactivation earlier and more frequently in comparison to those who received MP pulses (p = 0.125), as shown by the Kaplan–Meier plot in **Figure 3A**. AZA was more commonly used as induction therapy in controls (19.4% controls *vs.* 4.2% cases, p = 0.02), while cyclosporin (CsA) was more frequently employed in patients who developed HZ (10.4% cases *vs.* 1.1% controls, p = 0.02). The Kaplan–Meier plot in **Figure 3B** shows the development of HZ reactivation according to the ISs chosen for the induction. Although not statistically significant, patients treated with MMF and CsA tended to develop HZ reactivation earlier compared to those treated with CYC, AZA, and RTX.

**Table 2:** Comparison between demographic and clinical characteristics after matching of cases (patients who developed HZ) and controls (patients who did not develop HZ).

	HZ patients (cases) (n = 58)	Matched Controls (n = 116)	p
**Mean duration of SLE before LN diagnosis (months)**	3.5 ± 5.8	3.5 ± 6.2	0.52
**Age at LN diagnosis (years)**	29.0 (IQR 21.0–42.0)	28.5 (IQR 21.0–40.0)	0.91
**Sex (female)**	94.8% (55/58)	93.1% (108/116)	0.75
**Histological class**			
Proliferative forms (III and IV)	81.8% (45/55)	81.4% (92/113)	1.00
Non-proliferative forms (II and V)	18.2% (10/55)	18.6% (21/113)	
**Activity index**	6 (IQR 3–10)	6 (IQR 4–9)	0.85
**Chronicity index**	2 (IQR 1–3)	1 (IQR 0–3)	0.18
**Creatinine (mg/dL)**	0.88 (IQR 0.70–1.19)	0.90 (IQR 0.70–1.30)	0.74
**eGFR (mL/min)**	79.5 (IQR 45.5–101.0)	80.5 (IQR 57.8–103.7)	0.68
**eGFR > 60 mL/min and creatinine ≤ 1 mg/dL**	63.8% (37/58)	53.5% (62/116)	0.26
**Arterial hypertension**	43.1% (25/58)	50.0% (58/116)	0.42
**Proteinuria (g/day)**	4.0 (IQR 1.8–5.4)	3.6 (IQR 2.1–5.5)	0.87
**Nephrotic syndrome (proteinuria > 3.5 g/day)**	58.6% (34/58)	52.6% (61/116)	0.52
**Induction glucocorticoids**			
Methylprednisolone pulses	79.0% (45/57)	88.8% (95/107)	0.11
Oral glucocorticoids	21.0% (12/57)	11.2% (12/107)	0.44
**Hydroxychloroquine**	32.8% (19/58)	23.3% (27/116)	0.20
**Induction immunosuppressants**			
Cyclophosphamide	54.2% (26/48)	51.6% (48/93)	0.39
Azathioprine	4.2% (2/48)	19.4% (18/93)	**0.02**
Mycophenolate	18.8% (9/48)	21.5% (20/93)	0.33
Cyclosporin	10.4% (5/48)	1.1% (1/93)	**0.02**
Rituximab	12.4% (6/48)	6.4% (6/93)	0.18
**GCs cumulative dose (g)**	26.5 (IQR 14.9–65.3)	30.9 (IQR 14.5–59.2)	0.59 0.40
**GCs cumulative dose >50 g**	62.1% (36/58)	69.0% (36/116)	
**GCs cumulative dose >100 g**	6.9% (4/58)	6.0% (7/116)	1.00
**MMF cumulative dose (g)**	1958.6 ± 3122.3	1592.7 ± 2740.9	0.43
**MMF cumulative dose >1,000 g**	44.8% (26/58)	35.3 (41/116)	0.25
**CYC cumulative dose (g)**	8.3 ± 16.2	9.4 ± 34.4	0.82
**CYC cumulative dose >5 g**	41.4% (24/58)	37.1% (43/116)	0.62
**Follow-up duration (years)**	20.7 (IQR 9.4–31.0)	16.2 (IQR 7.0–25.4)	0.09
**CKD development**	10.3% (6/58)	16.5% (19/115)	0.36
**Death**	13.8% (8/58)	11.2% (13/116)	0.63

C3, complement factor 3; C4, complement factor 4; CKD, chronic kidney disease; CYC, cyclophosphamide; ENA, extractable nuclear antigen (anti-SSA [anti-Sjögren’s-syndrome-related antigen A autoantibodies], anti-SSB [anti-Sjögren’s-syndrome-related antigen A autoantibodies], anti-SM [anti-Smith antibodies], and anti-RNP [anti-negative antinuclear ribonucleoprotein antibodies]); HZ, herpes zoster; MMF, mycophenolate mofetil; WBC, white blood cells; GCs, glucocorticoids. Bold values used for statistically significant results.

### 3.6 Predictors of HZ occurrence at univariate and multivariate analyses

The univariate analysis (**Table 3**) identified that the use of CsA (p = 0.011) and more than 50 g of GCs (p = 0.004), 5 g of CYC (p = 0.001), and 1,000 g of MMF (p = 0.007) emerged as predisposing factors for the development of HZ. On the contrary, the use of MP pulses (p = 0.010) and AZA (p = 0.008) at induction seemed to exert a protective effect. The multivariate analysis (**Table 3**), although not statistically significant, suggested the protective effect of MP pulses and AZA at induction, while higher GCs and MMF cumulative dose seemed to predispose to HZ.

Table 3Univariate and multivariate logistic regression analyses for the risk of herpes zoster (HZ) development.

**Table:** 

Univariate analysis			
	**OR**	**95% CI**	**p**
**Activity index**	1.015	0.942–1.094	0.698
**Chronicity index**	1.050	0.893–1.235	0.555
**Creatinine**	1.001	0.731–1.380	0.979
**Proteinuria**	0.950	0.861–1.047	0.280
**C3**	0.996	0.983–1.008	0.512
**C4**	1.010	0.978–1.041	0.589
**Induction CS (IV pulses)**	0.474	0.197–1.137	**0.010**
**Induction with cyclophosphamide (CYC)**	1.382	0.755–2.530	0.291
**Induction with azathioprine**	0.181	0.040–0.817	**0.008**
**Induction with mycophenolate (MMF)**	0.730	0.295–1.806	0.490
**Induction with cyclosporin**	10.698	1.212–94.384	**0.011**
**Induction with rituximab**	2.071	0.630–6.809	0.234
**Hydroxychloroquine**	1.606	0.780–3.225	0.186
**CS cumulative dose (g)**	0.999	0.999–1.000	0.468
**CS cumulative dose >50 g**	1.358	0.701–2.628	**0.004**
**MMF cumulative dose (g) average**	1.000	0.999–1.000	0.434
**MMF cumulative dose >1,000 g**	1.486	0.782–2.826	**0.007**
**CYC cumulative dose (g) average**	0.999	0.999–1.000	0.809
**CYC cumulative dose >5 g**	1.198	0.629–2.282	**0.001**

**Table:** 

Multivariate analysis			
	**OR**	**95% CI**	**p**
**Induction corticosteroids (IV pulses)**	0.250	0.086–0.721	**0.008**
**Induction with azathioprine**	0.114	0.022–0.593	
**CS cumulative dose >50 g**	1.445	0.611–3.415	
**CYC cumulative dose >5 g**	0.942	0.435–2.040	
**MMF cumulative dose >1,500 g**	1.185	0.551–2.550	

C3, complement factor 3; C4, complement factor 4; CI, confidence interval; CS, corticosteroids; CYC, cyclophosphamide; IV, intravenous; MMF, mycophenolate; OR, odds ratio. Bold values used for statistically significant results.

## 4 Discussion

In our LN patients, we found a prevalence of HZ of 22.6% and an incidence of 15.6 per 1,000 person-years; these results are in keeping with those reported in SLE patients (7, 8, 14, 15) and significantly higher compared to those of the general population (incidence 1.2 to 4.9 per 1,000 person-years) (12, 13). Nevertheless, it is essential to acknowledge that the data could be influenced by the study’s retrospective nature, its prolonged duration, and the reliance on clinical reports for HZ diagnosis, potentially introducing biases. There are limited data about the differences in the epidemiology of HZ reactivation between patients with SLE and LN. However, two separate studies on SLE have observed that patients experiencing HZ reactivation have a higher likelihood of having previously encountered severe complications such as LN (14, 15). This finding may be explained by the severe immunosuppressive condition caused by the need for aggressive treatment in LN to induce remission (29) and by the impaired immunity that characterizes the active LN phases. In fact, it has been suggested that dysfunction in T cells, B cells, and NK cells may contribute to infection development in SLE patients (30). Contrary to expectations, we did not observe a higher incidence of HZ during the active phase of LN. Approximately half of the patients in our cohort developed HZ during a period of stable remission of LN, with about a quarter of them receiving no immunosuppressive therapy at the time. These findings suggest that other factors than immunosuppressive therapy and impaired immunological status may predispose HZ.

Our data show a progressive increase in HZ incidence during the last 50 years, with a peak incidence rate after 2010 of 43.9 per 1,000 person-years. These results, which should nevertheless be confirmed by larger and preferably prospective cohort studies, may suggest that HZ is a significant issue in LN patients. The increased HZ incidence during the last decades could be attributed to the increasing use of combined immunosuppressive drugs, including biological drugs, in the treatment of LN, as well as by the progressively more frequent use of ISs in maintenance therapy (29). As a matter of fact, treatment with high-dose GCs (7, 8, 18), ISs (14, 15), or concomitant use of both drugs (16) has been reported as risk factors for HZ in SLE patients (16). In a case–control study in which 65 SLE patients with HZ were matched with 130 SLE patients without HZ, ISs use and GC use emerged as risk factors for HZ (31). In the study by Mok et al., higher doses of prednisolone at induction therapy, peak daily MMF dose, and cumulative CYC dose during induction therapy were significantly associated with HZ reactivation in LN (8). Nonetheless, this study evaluated only the HZ risk during the first 2 years following the diagnosis of LN. A meta-analysis of 17 randomized controlled trials that evaluated the treatments of LN found that the risk of HZ was increased with the use of CYC, MMF, or tacrolimus in combination with GCs, compared with GCs alone (32). To thoroughly explore the role of the treatments in HZ, we matched the patients according to sex, age at diagnosis of LN, year of diagnosis of LN, and histological class at kidney biopsy to exclude confounding factors. At univariate analysis, the use of CsA and higher cumulative dosage of GCs, CYC, and MMF was associated with an increased risk of HZ reactivation. However, at multivariate analysis, CsA did not emerge as an independent predictor probably because of the small number of patients treated with this drug. At univariate analysis, the use of MP pulses was protective against HZ. This trend might be attributed to the fact that the oral GC doses following MP pulses are typically lower compared to the doses used when induction therapy commences with oral GCs. Patients treated with oral GCs as induction therapy showed, at the Kaplan–Meier curves, not only a higher but also an early occurrence of HZ than those receiving induction with MP pulses. Furthermore, we have found that the use of AZA during induction therapy may be protective against HZ at univariate analysis. Unfortunately, multivariate analysis was unable to identify independent predictors of HZ.

In addition to immunosuppression, in this study, we did not find other possible predictors of HZ reactivation among the clinical, histological, and immunological features at diagnosis of LN. Moreover, the fact that more than half of HZ cases occur after 5 years confirms that HZ reactivation is difficult to predict in LN. HZ usually has a mild clinical presentation (10); however, also severe complications may occur (11). Only two patients in our cohort had a severe form of HZ with sequelae. Therefore, considering the unpredictability of HZ occurrence and the possibility of a severe presentation, vaccination should be recommended. The effectiveness of HZ vaccines, notably the recombinant zoster vaccine, in preventing HZ has been demonstrated in both healthy older individuals and selected immunocompromised populations (33, 34). Kidney Disease: Improving Global Outcomes (KDIGO) guidelines already encourage vaccination in LN (35), and, more recently, ACR guidelines recommend vaccinations in subjects with rheumatic disease (36). Since in patients who are receiving strong immunosuppression there is a risk of infection after the administration of live attenuated vaccines and a possible reduction of immunogenicity of vaccines (37, 38), vaccination should be performed with recombinant zoster vaccine during periods of disease quiescence in which immunosuppression is minimal or before starting IS treatment.

A limitation of this study is the retrospective nature, and we cannot exclude that we may have underestimated the number of HZ cases in the early years of the study. Furthermore, we were unable to assess the real impact of hydroxychloroquine therapy on the potential development of HZ. In fact, not all our patients received hydroxychloroquine at the time of their LN diagnosis, as suggested by guidelines nowadays (3), especially for those diagnosed in the 1970s and 1980s. This could be attributed to the limited utilization of this medication in nephrology units during the early years of our study.

Finally, while the relatively small sample size in this study calls for multi-center studies with larger sample sizes to better explore the predisposing factors of HZ in LN, a strength of this study is the long follow-up of the patients, which allowed for analysis of the long-term outcome in patients with LN who developed HZ.

## 5 Conclusions

This study confirmed that HZ, with a prevalence of 22.6% and an incidence of 15.6 per 1,000 person-years, represents a more common complication among individuals with LN compared to the general population. Furthermore, with more than half of the cases occurring 5 years from the diagnosis of LN, regardless of the disease stages, HZ is an unpredictable complication. Taking into consideration these observations and the fact that severe forms are possible, vaccination should be advocated. However, establishing the timing of vaccination is not easy. If it has not been conducted before the diagnosis of LN, it is reasonable to suggest its use during a quiescent phase when the dose of immunosuppressants is minimal. The use of AZA and MP pulses at induction turned out to be protective. However, higher cumulative doses of CS, CYC, and MMF seemed to be predisposing factors. However, multi-center studies on a larger sample size are required to better explore the predisposing factors of HZ in LN and to develop a tailored approach to this complication.

## Data availability statement

The raw data supporting the conclusions of this article will be made available by the authors, without undue reservation.

## Ethics statement

The studies involving humans were approved by Ethics Committee of IRCCS Humanitas Research Hospital, Milano, Italy. The studies were conducted in accordance with the local legislation and institutional requirements. The participants provided their written informed consent to participate in this study.

## Author contributions

FR: Conceptualization, Data curation, Formal analysis, Investigation, Methodology, Visualization, Writing – original draft, Writing – review & editing. SC: Data curation, Investigation, Writing – review & editing. FT: Conceptualization, Data curation, Formal analysis, Writing – review & editing. MC: Investigation, Supervision, Writing – review & editing. LL: Investigation, Visualization, Writing – review & editing. MG: Conceptualization, Supervision, Writing – review & editing. NP: Conceptualization, Supervision, Writing – review & editing. GM: Conceptualization, Data curation, Investigation, Supervision, Writing – original draft, Writing – review & editing.
