# Supplementary material for: Herpes zoster in lupus nephritis: experience on 292 patients followed up for 15 years
Source: Front Immunol. 2023 Nov 22;14:1293269. doi: 10.3389/fimmu.2023.1293269 (PMC10703468; doi:10.3389/fimmu.2023.1293269)
Supplement: Supplementary file 1 [file Table_1.docx]

|  | **HZ patients (n=66)** | **Controls (n=226)** | **p** |
| --- | --- | --- | --- |
| **Duration of SLE before LN diagnosis (months)** | 3.3 ± 5.6 | 3.5 ± 6.0 | 0.88 |
| **Age al LN diagnosis (years)** | 31.28 (21.25-39.07) | 28.39 (21.20-39.74) | 0.35 |
| **Sex (female)** | 92.4% (61/66) | 88.5% (199/226) | 0.22 |
| **Histological Class** |  |  |  |
| Proliferative forms (III and IV) | 81.0% (51/63) | 74.5% (158/212) | 0.23 |
| Non proliferative forms (II and V) | 19.0% (12/63) | 25.0% (53/212) |  |
| Class VI | 0.0% (0/63) | 0.5% (1/212) |  |
| **Activity index** | 6 (IQR 3-10) | 6 (IQR 3-9) | 0.79 |
| **Chronicity index** | 2 (IQR 1-4) | 1 (IQR 0-3) | **0.03** |
| **Creatinine (mg/dL)** | 0.9 (IQR 0.7-1.4) | 0.9 (IQR 0.7-1.3) | 0.91 |
| **eGFR (mL/min)** | 73.8 (IQR 43.3-99.1) | 80.0 (IQR 55.9-111.1) | 0.17 |
| **eGFR > 60 mL/min and creatinine ≤ 1 mg/dL** | 36.4% (24/66) | 42.9% (97/226) | 0.39 |
| **Arterial hypertension** | 50.0% (33/66) | 50.2% (112/223) | 1.00 |
| **Proteinuria (grams/day)** | 4.0 (IQR 1.8-5.3) | 3.2 (IQR 2.0-5.4) | 0.94 |
| **Nephrotic syndrome (proteinuria > 3.5 grams/day)** | 56.1% (37/66) | 47.8% (108/226) | 0.26 |
| **Serum albumin (g/dL)** | 4.0 (IQR 1.8-5.3) | 3.2 (IQR 2.0-5.4) | 0.94 |
| **WBC (n/mmc)** | 5800 (IQR 4100-7600) | 5600 (IQR 3900-7490) | 0.63 |
| **Hemoglobin (g/dL)** | 11.3 (IQR 9.5-12.6) | 11.1 (IQR 9.5-12.3) | 0.58 |
| **Platelets (****10^3^/uL)** | 261 (IQR 190-352) | 219 (IQR 172-293) | **0.02** |
| **C3 (mg/dL)** | 54.0 (IQR 46.0-73.0) | 58.5 (IQR 48.0-79.0) | 0.41 |
| **C4 (mg/dL)** | 11.0 (IQR 5.5-14.6) | 9.8 (IQR 5-14) | 0.54 |
| **Anti-DNA positivity** | 100% (66/66) | 99.6 (225/226) | 1.00 |
| **Antiphospholipid antibodies positivity** | 38.7% (IQR 24/62) | 36.1% (69/191) | 0.76 |
| **ENA (****anti-SSA, anti-SSB, anti-SM and/or anti-RNP) positivity** | 62.3% (38/61) | 54.4% (92/169) | 0.30 |
| **Induction glucocorticoids** |  |  |  |
| Methylprednisolone pulses | 81.3% (52/64) | 83.3% (174/209) | 0.42 |
| Oral glucocorticoids | 18.8% (12/64) | 16.8% (35/209) |  |
| **Hydroxychloroquine** | 27.3% (18/66) | 22.6% (51/226) | 0.42 |
| **Induction immunosuppressor** |  |  |  |
| Cyclophosphamide | 54.7% (29/53) | 47.7% (83/174) | 0.89 |
| Azathioprine | 5.7% (3/53) | 15.5% (27/174) | 0.06 |
| Mycophenolate | 18.9% (10/53) | 24.7% (43/174) | 0.45 |
| Cyclosporin | 9.4% (5/53) | 3.5% (6/174) | 0.07 |
| Rituximab | 11.3% (6/53) | 8.6% (15/174) | 0.67 |
| **GCs cumulative dose (grams)** | 48.9 (IQR 20.6-87.3) | 21.5 (IQR 10.4-45.5) | **< 0.0001** |
| **GCs grams for year of follow-up** | 3.0 (IQR 1.2-5.3) | 1.9 (IQR 0.9-4.7) | 0.09 |
| **Follow-up (years)** | 19.6 (IQR 9.4-28.7) | 13.8 (IQR 5.2-24.0) | **0.005** |
| **CKD development** | 12.1% (8/66) | 20.6% (46/223) | 0.08 |
| **Death** | 13.6% (9/66) | 13.7% (31/226) | 1.00 |

***Supplemental Table 1.*** *Demographic and clinical characteristics of the lupus nephritis cohort. C3, complement factor 3; C4, complement factor 4; CKD, chronic kidney disease; GCs, corticosteroids; ENA, extractable nuclear antigen (anti-SSA [anti–Sjögren's-syndrome-related antigen A autoantibodies], anti-SSB [anti–Sjögren's-syndrome-related antigen A autoantibodies], anti-SM [anti-Smith antibodies], anti-RNP [anti negative antinuclear ribonucleoprotein antibodies]); HZ, herpes zoster; WBC, white blood cells.*
