# Supplementary material for: Herpes zoster in lupus nephritis: experience on 292 patients followed up for 15 years
Source: Front Immunol. 2023 Nov 22;14:1293269. doi: 10.3389/fimmu.2023.1293269 (PMC10703468; doi:10.3389/fimmu.2023.1293269)
Supplement: Supplementary file 2 [file Table_2.docx]

|  | **Overall** | **Active disease*** | **Inactive disease**** | **p** |
| --- | --- | --- | --- | --- |
| **Number of patiens** | 66 | 29 | 37 |  |
| **Corticosteroids (n, %)** | 55 (83.3%) | 26 (89.7%) | 29 (78.4%) | 0.224 |
| **Corticosteroids dose (mg/day, average ± SD)** | 10.5 ± 3.7 | 11.5 ± 7.0 | 6.2 ± 6.3 | 0.001 |
| **Hydroxychloroquine (n, %)** | 22 (33.3%) | 7 (24.1%) | 15 (40.5%) | 0.235 |
| **Immunosuppressors (n, %)** | 51 (77.3%) | 20 (69.0%) | 31 (83.8%) | 0.153 |

**Supplementary Table 2.** Comparison of treatments used in patients with active and inactive lupus nephritis at the time of herpes zoster infection.

*Active disease patients include 4 patients with nephritic flare, 10 patients with proteinuric flare, and 15 patients with partial remission.

**Inactive disease patients include 36 patients with complete remission and 1 patient with chronic kidney disease.
